# *Staphylococcus argenteus* as an etiological agent of prosthetic hip joint infection: a case presentation

**DOI:** 10.7150/jbji.44848

**Published:** 2020-05-25

**Authors:** Bo Söderquist, Peter Wildeman, Bianca Stenmark, Marc Stegger

**Affiliations:** 1 Department of Laboratory Medicine, Faculty of Medicine and Health, School of Medical Sciences, Örebro University, Örebro, Sweden.; 2 Department of Orthopedics, Faculty of Medicine and Health, School of Medical Sciences, Örebro University, Örebro, Sweden.; 3 Department of Bacteria, Parasites and Fungi, Statens Serum Institut, Copenhagen, Denmark.

**Keywords:** prosthetic joint infections, antimicrobial agents, genome analysis, *Staphylococcus argenteus*

## Abstract

This report presents a case of prosthetic hip infection caused by *Staphylococcus argenteus,* a potentially overlooked etiology of prosthetic joint infections (PJIs). Whole-genome sequencing showed that the *S. argenteus* isolate was an ST2250 and clustered within other CC2250 isolates, the largest clonal group of *S. argenteus*. This sequence type is prevalent and may be associated with invasive infections. The present isolate was phenotypically fully susceptible to all tested antimicrobial agents and genome analysis did not detect any resistance genes, nor were any staphylococcal cassette chromosome residues detected. Despite initial appropriate management with debridement and biofilm-active antibiotics, the outcome was unfavorable with recurrence and a persistent infection treated with suppressive antibiotics. Regarding the repertoire of genomic traits for virulence in *S. argenteus*, PJIs caused by this bacterium should be treated accordingly as *Staphylococcus aureus* PJIs.

## Introduction

*Staphylococcus aureus* is, together with *Staphylococcus epidermidis*, the most common cause of prosthetic joint infections (PJIs). *Staphylococcus argenteus* is a novel staphylococcal species closely related to *S. aureus* and considered a part of the *S. aureus* complex, which also includes *Staphylococcus schweitzeri* 1,2,3.

The geographical distribution of this species is unknown although many of the early reports originate from Africa, Asia and Australia/New Zealand 4,5,6,7. From Europe the reports are sparse; 25 *S. argenteus* genomes from Denmark 8, low prevalence (0.16%) in a nationwide Belgian study 9, a case report from France 10 and one strain from UK 3. However, among isolates classically identified as methicillin-resistant *S. aureus* (MRSA) in Sweden that is normally considered a low prevalence country regarding MRSA, *S. argenteus* has also been identified 11,12.

The clinical features of *S. argenteus* infections are infrequently described 1 but include bacteremia 13, skin and soft tissue infections 8,9 and a bone and joint infection 10. Hitherto, only one case of a prosthetic hip infection, from China, has been described previously 14. In a retrospective study of PJIs caused by *S. aureus* we found one out of 101 cases to be caused by *S. argenteus* following whole-genome sequencing. The aim of this report was to describe this case of PJI caused by *S. argenteus,* a potential overlooked etiology of PJIs.

## Case presentation

The patient was a 70-year-old woman with type 2 diabetes mellitus, Alzheimer's dementia, previous alcohol abuse, and suspected liver cirrhosis. She had multiple wounds on her legs due to suspected vasculitis.

The patient contracted a dislocated femoral neck fracture of the right hip on 6th of October 2015 (day 1) and received a total hip replacement two days later. The post-operative course was uneventful, but the patient experienced increasing pain of the hip and finally redness during the middle of November where swelling of wound area and also fever was noted. At admission on day 23 the body temperature was 39.0 °C, blood pressure 95/60, and saturation 84%. The C-reactive protein (CRP) was measured at 277 mg/L (normal range <4 mg/L). Empirical antibiotic treatment with cefotaxime and gentamicin was instituted. However, ultrasound-guided arthrocentesis and blood cultures grew *S. aureus* identified by MALDI-TOF MS (Microflex LT and Biotyper 3.1, DB5989, Bruker Daltonik, Bremen, Germany) and antibiotic treatment was changed to cloxacillin 2 g tid intravenously. The tested isolates were all fully susceptible according to EUCAST break points (http://www.eucast.org) to cefoxitin, fusidic acid, clindamycin, gentamicin, rifampicin, trimethoprim-sulphamethoxazole, ciprofloxacin, linezolid (MIC 1 mg/L), daptomycin (MIC 0.19 mg/L) and vancomycin (MIC 1 mg/L). Debridment and irrigation was performed at day 25 and modal components were exchanged but the implant was retained (DAIR). Cultures from five tissue biopsies were also positive with growth of *S. aureus*. After seven days of intravenous treatment with cloxacillin the treatment was changed (day 32) to oral rifampin 300 mg bid and ciprofloxacin 750 mg bid and the patient was discharged. However, the patient was readmitted due to nausea and a renal insufficiency was noted why ciprofloxacin was discontinued. A combination of rifampin and fusidic acid was attempted but was not either later on tolerated by the patient why suppressive treatment with flucloxacillin was chosen. At a follow-up visit after three months the functional status was rather good and the patient was mobilized with a walker without any significant pain. However, the CRP was still elevated, 23 mg/L, and according to the insufficient eradication treatment completed, continuous suppressive treatment with flucloxacillin was recommended. Despite that antimicrobial treatment was discontinued five months later. The patient was then re-admitted after one additional month and cultures both from the synovial fluid and the blood displayed growth of *S. aureus.* No DAIR was performed and after i.v. treatment with cloxacillin suppressive treatment with flucloxacillin was re-instituted.

**Figure 1:**
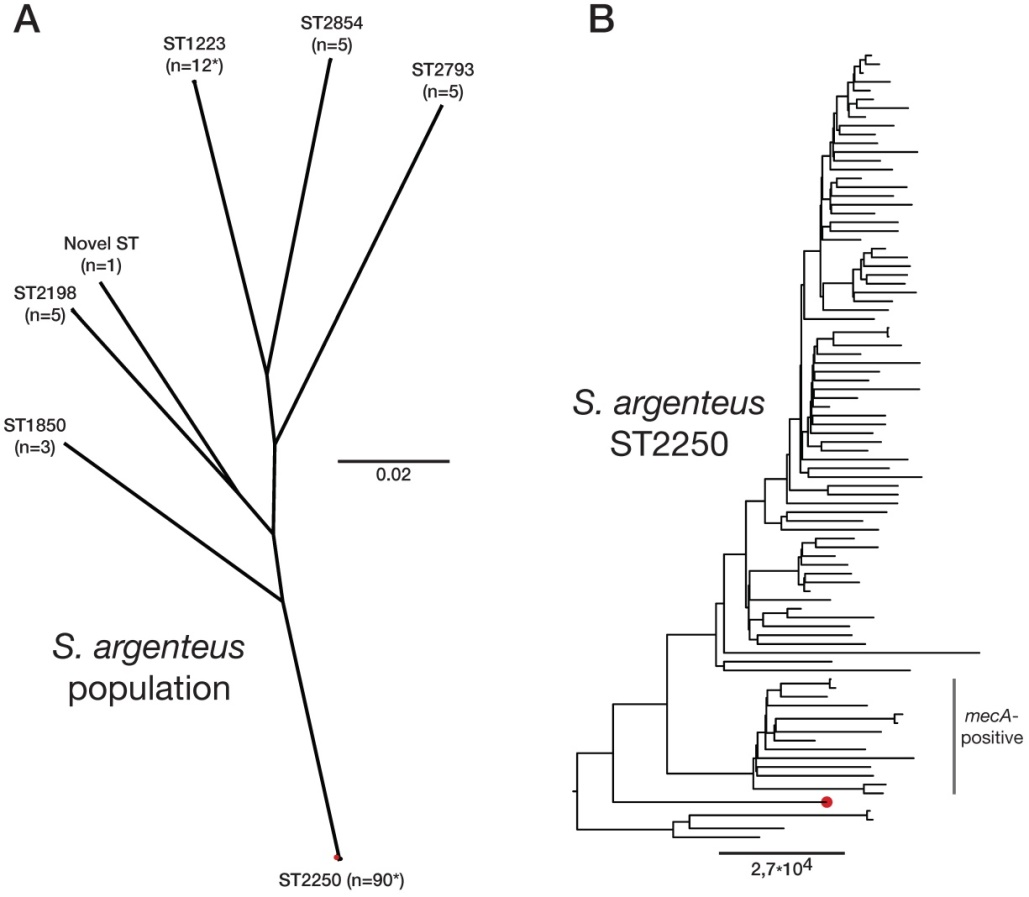
** Phylogenetic clustering of *Staphylococcus argenteus*.** (**A**) Unrooted phylogeny based on 52,329 SNPs depicting all available *S. argenteus* genome sequences available in the NCBI Reference Sequence database (n=120) and the isolate from this study. Sequence types (STs) and number of isolates per clade is highlighted with * indicating the presence of other STs. (**B**) Detailed rooted phylogeny of the ST2250 clade where MRSA isolates are noted as are the Swedish PJI isolate in red. Scalebar indicate substitutions per site.

In a retrospective study of PJI caused by *S. aureus*, all isolates were whole-genome sequenced 15. However, the PJI isolate from the present patient turned out to cluster distinct of the other isolates (data not shown) and to be of sequence type 2250 which combined identified it as *S. argenteus*. A maximum-likelihood phylogenetic analysis using IQ-TREE of all publically available *S. argenteus* genomes (n=120) from NCBIs RefSeq database confirmed this and it clustered as expected within the ST2250 clade, **Figure 1**. Analyses of resistance markers using ResFinder v3.1 (https://cge.cbs.dtu.dk/services/ResFinder) found that no such was present. This is accordance to the result of the antibiotic susceptibility test, see above. Investigation of presence of virulence genes using VirulenceFinder (http://www.genomicepidemiology.org) showed >99% identity for *scn* and *sak* and > 85% indentity was found for *aur*, *hlgB* and *lukE*, all against *S. aureus* gene variants. The genome sequence data is available from the Sequence Read Archive (SRA) ID PRJEB36681 with read accesion ID ERR3890992.

## Discussion

Staphylococci are by far the most common causes of PJIs. Almost half of all staphylococcus-associated PJIs are caused by *S. aureus,* both early post-interventional infections and late, but often acute, haematogenous infections. *S. argenteus* is a coagulase-positive staphylococcus first described in 2006 16. Although rare 9, it may have been overlooked since the species determination using routine methods at clinical microbiological laboratories have been challenging without whole-genome sequencing 17. However, MALDI-TOF have become a useful tool for accurately distinguish* S. argenteus* from* S. aureus* 13. The updated and improved database of Bruker Daltonik in October 2018 may in the future contribute to reveal the true incidence of serious infections, including PJIs, which could be assigned to *S. argenteus* instead of *S. aureus.* However, *S. argenteus* has been regarded as a low virulent species within *Staphylococcus aureus*-related complex 2,16. Nevertheless, there is an accumulation of reports on serious invasive infections including not only skin and soft tissue infections but also necrotizing fasciitis 2,4, bone and joint infections 5,10,14,18 and bacteremia 13. In addition, the only case of a PJI reported so far concerns a patient suffering from a persistent and recurrent infection in which a *S. argenteus* strain and its small colony variants (SCVs) was isolated 14. Furthermore, increased mortality among patients with bacteremia due to *S. argenteus* compared to MSSA has also been reported 13 despite susceptibility to most antibiotics.

The *S. argenteus* isolate described in this report was also obtained from a patient with a prosthetic hip infection and whole-genome sequencing showed that this isolate was an ST2250 and clustered within other CC2250 isolates, the largest clonal group of *S. argenteus*. Two recent studies have shown that ST2250 *S. argenteus* is highly prevalent, and may be associated with invasive infections 5,7. The present isolate was phenotypically fully susceptible to all tested antimicrobial agents and genome analysis did not detect any resistance genes (including *blaZ*), nor any staphylococcal cassette chromosome residues. A systematic investigation of genome sequences encoding virulence genes has shown that *S. argenteus* harbor all virulence genes required for the pathogenicity in *S. aureus* 19, including the *ica* operon. Identification of virulence factors including the human phiSa3 prophage and carriage of *aur* encoding aureolysin has been linked to prothrombin activation and immune evasion 20. Our isolate displayed > 85% indentity only for *scn, sak, aur*, *hlgB* and *lukE* 19.

There was no indication that SCVs were identified according to the microbiological protocol or report, however, the PJI persisted which also may have been explained by the lack of efficient long-term biofilm-active treatment including rifampicin.

*S. argenteus* as an etiological agent of PJI may have been overlooked, especially if not displaying methicillin resistance. Regarding the repertoire of genomic traits for virulence in *S. argenteus,* PJIs caused by this bacterium should be treated accordingly as *S. aureus* PJIs.
